# Recognizing and Responding to the Commonly Misunderstood Reactions to Sexual Assault: Evaluation of an Online Curriculum

**DOI:** 10.1089/whr.2020.0062

**Published:** 2020-09-03

**Authors:** Robin Mason, Janice Du Mont, Stephanie Lanthier, Sheila Macdonald, Ilene Hyman

**Affiliations:** ^1^ Women's College Research Institute, Women's College Hospital, Toronto, Canada.; ^2^ Dalla Lana School of Public Health, University of Toronto, Toronto, Canada.; ^3^ Ontario Network of SA/DVTCs, Toronto, Canada.

**Keywords:** sexual assault, training, curriculum, health providers, evaluation

## Abstract

***Background:*** Sexual assault remains a serious public health issue with significant impacts on the health and well-being of individual women. Many women's reactions and behaviors post sexual assault are not well understood by the general public, or more worrying, among professionals to whom women frequently turn to for help. An innovative and evidence-informed online curriculum was developed to educate health and social service providers about the range of possible psychological responses and associated behaviors post sexual assault and to better equip them in supporting survivors in their recovery.

***Methods:*** The curriculum was evaluated using pre- and post-training tests designed to measure changes in fact-based knowledge, self-assessed knowledge, and procedural knowledge, that is, perceived competency.

***Results:*** A total of 759 participants registered to complete the curriculum between July 2018 and July 2019 and 175 completed both the pre- and post-training surveys. Data analyses showed significant improvement in the mean number of correct answers to the fact-based knowledge, self-reflection, and procedural knowledge questions from pre- to post-training. The response to the training was also very positive.

***Conclusions:*** The online curriculum was effective in improving participants' knowledge about and response to women who, in the aftermath of a sexual assault, may exhibit reactions or behaviors that are commonly misunderstood.

## Introduction

Sexual assault remains a serious global public health issue with enormous impacts on the health and well-being of women.^1^ Fanflik^2^ writes, “As there is nothing normative about being sexually victimized, there cannot be a ‘normal’ reaction to such a traumatic event” (p. 9). This is a particularly important observation as recent media attention has highlighted gaps in our collective understanding of the psychological effects of sexual assault apart from post-traumatic stress disorder (PTSD), anxiety, and depression. A sexually assaulted woman may appear tearful, or quiet and contained, express anger, or be in shock. She may be in denial about the sexual assault, or experience confusion, panic, shame, revulsion, guilt, humiliation, and low self-esteem, leading to self-isolation.^3^

Expectations about how a sexually assaulted woman should react have continued to influence responses to disclosure, often serving to reinforce misguided beliefs about who is considered a legitimate or “real” victim.^4^ Behaviors such as acting as though everything is fine or convincing oneself that “the assault could have been worse,” seeking an explanation from the perpetrator, or maintaining the relationship after an assault, may confuse health and social service providers who may respond with disbelief and judgmental comments, reactions that further traumatize the survivor, sabotaging the already complex healing process.^5^

Studies on the process of disclosure in the aftermath of sexual assault, reactions to disclosure, and the impact of the varied reactions to disclosure on survivors' coping, help-seeking, and healing, have demonstrated the significant impact of both positive and negative responses to survivors' recovery and well-being.^6^ Stigmatizing comments and high levels or prior trauma have been identified as exacerbating post-traumatic stress reactions and heightening the risk of PTSD.^7^ Survivors have particularly commented on their frustration with the “unhelpful” responses and lack of “tangible aid” provided by formal providers, including nurses, physicians, and social workers.^8^

Health and social service providers often lack the requisite knowledge and skill to recognize and respond in a positive way to disclosures of sexual assault,^9–11^ particularly when survivors present with unexpected behaviors or reactions. To address this gap, our research team, with extensive expertise in developing and evaluating curricula related to interpersonal violence,^12–15^ and aided by external experts, developed and evaluated a competency-based, evidence-informed online curriculum designed to educate health and social service providers about the range of possible reactions and associated behaviors of survivors in the aftermath of a sexual assault.

Competency-based education and training align educational content with the specific needs of the population and system. The origins of competency-based education can be traced back to the 1960s and 1970s when teachers began to focus on students' achievement of specific objectives rather than the time they spent in learning or teachers in teaching (see, *e.g.*, Spady and Mitchell^16^). Thus, competency-based education is outcome based; the requisite knowledge, skills, and abilities to achieve specific outcomes are identified and individual learners supported to acquire these at their own pace. More recently, these methods have been adopted in the preparation and continuing education of medical and health professionals.^17–19^

## Development of the Curriculum

The curriculum was developed to challenge any negative attitudes held by health or social service providers while promoting the essential knowledge and skills needed to appropriately care for survivors of sexual assault. In designing the format for the curriculum, we drew upon social cognition theory and its recognition that new behaviors are acquired through the dynamic and reciprocal interaction of the individual, external social context, and achievement of specific competencies.^20^

We invited a group of external experts to form an advisory committee to help with this project. Members included those with “lived experience,” physicians, nurses including sexual assault nurse examiners from both urban and more rural settings, and allied health and social service professionals. Members had extensive expertise in the delivery of health and social services to women who had experienced sexual assault as well as other forms of gender-based violence, and represented a range of disciplines and philosophical perspectives. The advisory committee members provided essential real-world experiential knowledge to the development of curriculum competencies and content.

Using a rigorous competency and evidence-informed approach, and following a method developed for other online trainings,^21–23^ the curriculum's objectives were articulated as follows:

- challenge rape myths and negative attitudes held about sexual assault victims;
- introduce health and social service providers to the full range of survivors' reactions in the aftermath of sexual assault, including the neurobiology involved; and
- promote appropriate supportive responses by providers to survivor behaviors that may be commonly misunderstood.

We initially searched the scholarly and gray literature using terms such as normalization, minimization, dramatization, suppression, explanation, and flight from the sexual assault, terms drawn from rape trauma syndrome's “outward adjustment stage,” identified in 1974 by Burgess and Holmstrom^24^ in their seminal work on traumatic reactions to sexual assault. Rape trauma syndrome is still referenced when describing survivors' psychological reactions to sexual assault.^25^ With input from the advisory committee, the search terms were expanded to include for example, numbing, dissociation, disinhibition, and emotional dysregulation and terms associated with sociocultural factors that potentially influence survivor reactions (*e.g.*, race, poverty, and disability) (see Appendix A1 for a list of search terms).

To complete the review of the scholarly literature, we searched OVID Medline, PsycINFO, and Social Work Abstracts databases. For the gray literature, we searched the National Clearinghouse Guidelines, as well as Google. Inclusion criteria were English language, sexual assault of adult or adolescent, women-identified victim/survivor, and reference to a commonly misunderstood reaction (*e.g.*, minimizing the sexual assault, not acknowledging the sexual assault, and increase in sexual activity after being sexually assaulted). The searches were conducted in November 2016 with no limitations applied to publication date.

The searches of the scholarly literature resulted in 183 publications, while the search of the gray literature yielded 84 items. After deleting duplicates and applying our inclusion criteria, the final sample comprised 59 items, including scholarly journal articles (*n* = 52), an edited book chapter (*n* = 1), and handbooks, reports, or videos (*n* = 6). The commonly misunderstood reactions and recommendations on how to recognize and respond (if these were noted) were extracted, organized, and uploaded to an online database developed for this project.

During a half-day meeting, the advisory committee reviewed and confirmed the relevancy of these materials in supporting clients after a sexual assault. As well, members highlighted significant critical gaps in the literature.

The identified gaps included the type of perpetrator as an influence on the survivor's reaction (*e.g.*, intimate partner versus stranger); the ways in which substance use during the assault can impact the survivor's experience, presentation to a health or social service provider, and the provider's response; intersecting forms of identity as an influence on survivor and provider behaviors; and the most appropriate and beneficial ways for providers to help when faced with the wide range of survivor reactions or behaviors. Owing to the paucity of available literature on this last point, the committee's input was particularly valuable and considerable time was allocated to discussing the provision of sensitive and competent care in response to the full range of survivors' potential reactions.

Once consensus was reached on the requisite knowledge, practices, and behaviors providers need to understand the range of survivors' reactions, these were articulated as competencies.

The next step in curriculum development was to sort and group the competencies into clusters following the updated version of Bloom's taxonomy, “know (factual knowledge), know oneself (self-reflection), know how (procedural knowledge)” (see Appendix A2 for curriculum competencies). Using the competencies to inform the content, a text-based draft of the curriculum was developed, reviewed by the advisory committee, and their feedback integrated.

Once the competencies were approved, novel content in the form of actual newspaper headlines; video-taped lectures; and realistic case studies including a sexually assaulted sex worker living in poverty, a young racialized woman in a dating relationship, and a middle-aged economically privileged white woman sexually assaulted by her abusive husband, were developed. In addition, our definition of women was inserted early in the curriculum. The definition indicates that “women” refers to “those that self-identify as ‘women’, and may or may not include transgender women and non-binary persons. Where research and statistics have specifically included transgender and non-binary persons, this has been noted.”

Quizzes to assess learner progress and questions to inspire self-reflection were added to promote mastery of the competencies. Self-reflection questions challenged learners to consider how racism, sexism, and ableism might influence their reactions to and care of women from marginalized communities, including sex workers and Indigenous women. Interactive elements and links to external resources were also provided to supplement the learning, enhance learner engagement, and achievement of the curriculum objectives.

The final curriculum required approximately an hour and a half to complete, although following the many external links adds time to that estimate. The completed curriculum was uploaded to a designated website in July 2018. Although the curriculum has general applicability, due to funding restrictions, dissemination activities focused on potential user communities in the province of Ontario.

**FIG. 1.**
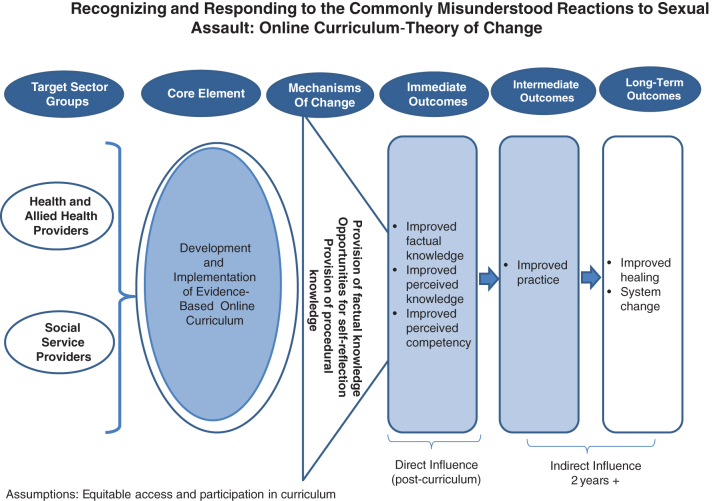
Theory of change.

As indicated earlier, the curriculum was primarily developed for health and social service providers but open to other types of providers. The mechanisms of change specified that the provision of factual knowledge regarding commonly misunderstood reactions to sexual assault, opportunities for self-refection, and the provision of procedural information on how to best respond in cases of sexual assault, would result in positive outcomes for participants and by extension, their clients. The proposed outcomes were identified for immediate (post-training), intermediate, and long-term intervals. The theory of change for the intervention is described in Figure 1.

## Evaluation of the Curriculum

The evaluation of the curriculum focused on the immediate outcomes described in the theory of change model. Before accessing the curriculum, learners registered and completed a pretest to assess factual knowledge, self-reflection of their own abilities, and procedural knowledge to determine how much they knew about how to respond to sexual assault. The same areas were assessed post-training along with items to assess satisfaction with, and the impact of, the training overall.

Within the factual knowledge domain are 12 general questions in the form of true/false, multiple choice, and fill in the blanks [*e.g.*, “Stereotypical gender norms and rape myths contribute to the mistreatment of sex workers who are sexually assaulted” (True/False)]. The self- knowledge domain asks the learner to engage in self-reflection and rate this by indicating their level of agreement on 10 statements (*e.g.*, “I am able to describe the effects of trauma on potential re-victimization”). Within the procedural competency domain are nine items in which learners indicate their level of agreement with statements such as “I know how to respond to survivors who have experienced (re)victimization” and “I can modify service provision to make it more accessible and comfortable for diverse clients.”

Table 1 describes the content of the factual, self-reflection, and procedural knowledge scales used to measure study outcomes including each of the individual items included within each scale.

**Table 1.** Outcome Scales and Items Assessed

Factual knowledge scale	1. ___________________ describes the ways that social factors (*e.g.*, racism, sexism, and ableism) interconnect and interlock to create a diverse range of experiences for individuals. As a result, the experience of sexual assault will differ from woman to woman.
	2. A person can consent to one form of sexual activity but not another, and can change their mind about having sex after providing consent. (True/False)
	3. Women with disabilities are no more or less likely to be sexually assaulted than women without disabilities. (True/False)
	4. Which of the following outcomes has been associated with sexual assault? (multiple choice)
	5. Women who have been sexually assaulted may not be able to fully describe what happened due to: (multiple choice)
	6. In comparison with women who have not been sexually assaulted, a woman who has been sexually assaulted is _______________________ to be sexually assaulted again in the future.
	7. Women may react in the following ways post sexual assault: (multiple choice)
	8. Which of the following reflects a woman's attempts to normalize the sexual assault? (multiple choice)
	9. Which of the following reflects a woman's attempts to explain why the sexual assault occurred? (multiple choice)
	10. Unacknowledged sexual assault is more likely to occur when the perpetrator is an intimate partner rather than a stranger. (True/False)
	11. In a ___________________ approach the service provider considers the impacts of systemic discrimination, such as sexism, racism, colonialism and ableism, alongside individual experiences of sexual assault (multiple choice).
	12. Which of the following are supportive responses for a client who may not acknowledge that she has been sexually assaulted? (multiple choice)
Self-reflection scale	1. I understand the problem of sexual assault in Canada
	2. I know how to recognize rape myths
	3. I can identify factors such as racism, sexism, ableism, and others that intersect to affect the experience of sexual assault, access to and care provided by health and social services
	4. I can articulate challenges associated with the issue of consent to sexual activity
	5. I can describe how the broader social context can shape individual biases that can impact the provision of care
	6. I can list the potential physical, social, emotional, and psychological outcomes of sexual assault
	7. I understand the neurobiological response to the trauma of sexual assault
	8. I am able to describe the effects of trauma on potential revictimization
	9. I can discuss commonly misunderstood reactions to sexual assault such as normalization, minimization, explanation, and dramatization
	10. I understand why sexual assault may be unacknowledged by the survivor
Procedural knowledge scale	1. I know how to respond to survivors who have experienced (re) victimization
	2. I am able to create safe and supportive spaces for disclosure
	3. I can modify service provision to make it more accessible and comfortable for diverse clients
	4. I am able to provide information about local resources and appropriate referrals
	5. I know how to respond when I believe a sexual assault has occurred but the woman has not acknowledged the experience as a sexual assault
	6. I am able to recognize commonly misunderstood reactions to sexual assault such as normalization, minimization, explanation, and dramatization
	7. I am able to create a safe and supportive environment for disclosure
	8. I am able to respond to a woman who has experienced (re) victimization
	9. I am able to respond to a woman who may not acknowledge her experience as a sexual assault

## Plan of Analysis

The data were imported into SPSS where they were reviewed and cleaned. Learners who completed both pre- and post-test surveys were matched. Descriptive statistics were used to create a demographic profile of the matched participants. Scales were created to represent the key outcomes of interest—factual knowledge, self-reflection, and procedural knowledge. The reliability of the three scales was assessed using Cronbach's α. The scores for each scale were calculated based on the number of correct responses for each of the scales. Paired *t*-tests were used to compare the mean number of correct responses among participants on the pre- and post-test scales.

## Results

### Description of participant learners

There were 759 learners who registered for the online curriculum between July 2018 and July 2019. Among the 175 learners who completed both the pre- and post-training tests, the majority identified their sex as female (89.73%) and their gender as a woman (87.9%). Two-thirds (67.2%) of the learners were between the ages of 25–54 years. Most indicated they worked in community service (36.8%) and health (28.2%), followed by education (6.9%), law enforcement/legal (5.2%), and government (3.4%) sectors. The majority worked in frontline positions (57.5%) and 21.9% were students/trainees. Over half of the learners (55.2%) had been practicing for 5 years or less, whereas 9.8% had worked between 6 and 10 years, and 33.3% had been working in their fields for 11 or more years.

### Improvements in factual knowledge, self-reflection, and procedural knowledge

Among learners, there was significant improvement in the mean number of correct answers to the factual knowledge questions pre- and post-training. There was also a significant increase in the number of self-reflection knowledge statements learners agreed with pre- and post-training and in the mean number of procedural knowledge statements learners agreed with pre- and post-training (Table 2).

**Table 2.** Comparisons of Pre- and Post-Test Scale Scores

Scale	Cronbach's α	Pre-test mean score	Post-test mean score	t*-Score (*p)
Factual knowledge	0.333	8.9	10.2	−11.25 (<0.000)
Self-reflection	0.858	7.9	9.6	−8.23 (<0.000)
Procedural knowledge	0.867	6.3	8.5	−10.5 (<0.000)

Those who completed the evaluation of the curriculum were very positive in their reviews. Almost all participants reported that they were satisfied with the quality of the curriculum (95%) and its format (92.1%). A very high proportion also reported that the curriculum was relevant to their work/profession (95.7%), increased their understanding of sexual violence (89.9%), increased their understanding of how to support someone who experienced sexual violence (89.9%), and enabled them to apply the knowledge to support someone who experienced sexual violence (92.8%). A very high proportion of participants (95.7%) also indicated that they would recommend this training to others.

## Discussion

Sexual assault is a ubiquitous form of gender-based violence with vastly more women than men victimized. Women who have been sexually assaulted can experience a range of significant negative physical, psychological, social, and emotional reactions. Although distress, anxiety, fear, and even PTSD are familiar anticipated reactions to sexual assault, there exist other commonly misunderstood, but expected, reactions. Service and health providers, as well as family and friends, may respond with negative, critical, or judgmental comments to these other behaviors and emotions. Negative responses to disclosure are associated with survivors' increased feelings of self-blame, shame, and isolation,^26^ which can lead to avoiding seeking other help, which can ultimately impact healing and recovery.^5,6,27^

The online curriculum was developed in response to the frequently experienced poor treatment received by women seeking care for sexual assault from health and social service providers. Interestingly, ∼15% of learners were from other than the targeted sectors. The curriculum challenges the many myths and misconceptions about the ways that survivors respond in the aftermath of sexual assault by detailing a full range of trauma-related behaviors, in the belief that with knowledge would come improved understanding and eventually more appropriate practice. The evaluation evidence suggests that the curriculum resulted in positive changes in knowledge, perceived knowledge, and competency among curriculum participants.

Some limitations to this evaluation are noted. First, although the curriculum was disseminated through multiple channels, we do not have data on who did not access the course and why. Second, the statistical reliability of the factual knowledge scale was low that may have been due to the questions that mixed multiple choice and short answer questions. Third, a sizable proportion of participants who registered for the online curriculum and completed the pre-test exited the program without completing the post-training evaluation. Finally, evidence of improved factual knowledge, self-reflection, and procedural knowledge does not necessarily translate into improved practice in the intermediate and longer term timeframe. Future research is necessary to examine whether improved professional response to survivor's disclosures contributes to their healing from sexual assault.

## Abbreviation Used

PTSD: post-traumatic stress disorder

## Appendix

### Appendix A1. Search Terms

(sexual assault or rape or sexual violence or sexual victimization or assault)
(behavior* or behavior* response* or behavior* reaction* or psychological response* or psychological reaction* or psychological consequence* or psychological impact*)
(trauma* or posttrauma* or post-trauma*)
(normaliz* or minimiz* or dramatiz* or suppress* or explain* or explanation or flight or dissocia* or disinhibit* or numb* or dysregulat*)
(counterintuitive or unacknowledged*)
(revictim* or traumatic bond or Stockholm syndrome)
(disab* or sexual orientation or sexual minority or race or cultur* or immigrant* or refugee* or indigenous or American Indian or native or metis or black or African American or poverty or socioeconomic or marginaliz*)

## Appendix A2: Curriculum Competencies

1. Recognize the problem of sexual assault in Canada
2. Understand the complexity of sexual assault within the context of individual and social experience (Describe societal factors)
3. a. Identify rape myths situated in the broader social context
4. b. Reflect on how social factors such as racism, sexism, ableism, colonialism, and others intersect, affecting the experience of sexual assault, access to, and care provided by services
5. c. Describe challenges associated with the issue of consent
6. d. Recognize how the broader social context can shape individual biases that can impact the provision of care
3. Understand the (neurobiology of) trauma of sexual assault
8. a. Recognize the physical, social, emotional, and psychological outcomes of sexual assault
9. b. Describe the neurobiological response to trauma
10. c. Recognize the potential effects of trauma for revictimization
4. Understand and identify commonly misunderstood reactions to sexual assault. Explain why sexual assault may be unacknowledged by the survivor.
5. Know how to respond to commonly misunderstood reactions to sexual assault. Be able to:
13. - create safe and supportive spaces for disclosure
14. - modify service provision to make it more accessible and comfortable for diverse clients
15. - respond to survivors who have experienced (re)victimization
16. - provide information about local resources and appropriate referrals
17. - respond to survivors who may not acknowledge their experience as sexual assault.
6. Reflect on your own beliefs, values, and practices
19. a. Recognize how your values, attitudes, beliefs, and experiences may shape and influence your response to survivors of sexual assault.
20. b. Examine your emotional responses to the commonly misunderstood behaviors of survivors of sexual assault.
